# Advances in osseointegration of biomimetic mineralized collagen and inorganic metal elements of natural bone for bone repair

**DOI:** 10.1093/rb/rbad030

**Published:** 2023-04-18

**Authors:** Wenbo Zhu, Chao Li, Mengxuan Yao, Xiumei Wang, Juan Wang, Wei Zhang, Wei Chen, Hongzhi Lv

**Affiliations:** Department of Orthopaedic Surgery, The Third Hospital of Hebei Medical University, Shijiazhuang 050051, P.R. China; Key Laboratory of Biomechanics of Hebei Province, Orthopaedic Research Institution of Hebei Province, Shijiazhuang 050051, P.R. China; NHC Key Laboratory of Intelligent Orthopaedic Equipment, The Third Hospital of Hebei Medical University, Shijiazhuang 050051, P.R. China; Department of Orthopaedic Surgery, The Third Hospital of Hebei Medical University, Shijiazhuang 050051, P.R. China; Key Laboratory of Biomechanics of Hebei Province, Orthopaedic Research Institution of Hebei Province, Shijiazhuang 050051, P.R. China; NHC Key Laboratory of Intelligent Orthopaedic Equipment, The Third Hospital of Hebei Medical University, Shijiazhuang 050051, P.R. China; Department of Orthopaedic Surgery, The Third Hospital of Hebei Medical University, Shijiazhuang 050051, P.R. China; Key Laboratory of Biomechanics of Hebei Province, Orthopaedic Research Institution of Hebei Province, Shijiazhuang 050051, P.R. China; NHC Key Laboratory of Intelligent Orthopaedic Equipment, The Third Hospital of Hebei Medical University, Shijiazhuang 050051, P.R. China; State Key Laboratory of New Ceramics and Fine Processing, School of Materials Science and Engineering, Tsinghua University, Beijing 100084, P.R. China; Department of Orthopaedic Surgery, The Third Hospital of Hebei Medical University, Shijiazhuang 050051, P.R. China; Key Laboratory of Biomechanics of Hebei Province, Orthopaedic Research Institution of Hebei Province, Shijiazhuang 050051, P.R. China; NHC Key Laboratory of Intelligent Orthopaedic Equipment, The Third Hospital of Hebei Medical University, Shijiazhuang 050051, P.R. China; Department of Pathology, Hebei Key Laboratory of Nephrology, Center of Metabolic Diseases and Cancer Research, Hebei Medical University, Shijiazhuang 050017, P.R. China; Department of Orthopaedic Surgery, The Third Hospital of Hebei Medical University, Shijiazhuang 050051, P.R. China; Key Laboratory of Biomechanics of Hebei Province, Orthopaedic Research Institution of Hebei Province, Shijiazhuang 050051, P.R. China; NHC Key Laboratory of Intelligent Orthopaedic Equipment, The Third Hospital of Hebei Medical University, Shijiazhuang 050051, P.R. China; Department of Orthopaedic Surgery, The Third Hospital of Hebei Medical University, Shijiazhuang 050051, P.R. China; Key Laboratory of Biomechanics of Hebei Province, Orthopaedic Research Institution of Hebei Province, Shijiazhuang 050051, P.R. China; NHC Key Laboratory of Intelligent Orthopaedic Equipment, The Third Hospital of Hebei Medical University, Shijiazhuang 050051, P.R. China

**Keywords:** mineralized collagen, bone defect reconstruction, inorganic metal elements, clinical applications

## Abstract

At this stage, bone defects caused by trauma, infection, tumor, or congenital diseases are generally filled with autologous bone or allogeneic bone transplantation, but this treatment method has limited sources, potential disease transmission and other problems. Ideal bone-graft materials remain continuously explored, and bone defect reconstruction remains a significant challenge. Mineralized collagen prepared by bionic mineralization combining organic polymer collagen with inorganic mineral calcium phosphate can effectively imitate the composition and hierarchical structure of natural bone and has good application value in bone repair materials. Magnesium, strontium, zinc and other inorganic components not only can activate relevant signaling pathways to induce differentiation of osteogenic precursor cells but also stimulate other core biological processes of bone tissue growth and play an important role in natural bone growth, and bone repair and reconstruction. This study reviewed the advances in hydroxyapatite/collagen composite scaffolds and osseointegration with natural bone inorganic components, such as magnesium, strontium and zinc.

## Graphical abstract

**Figure:**
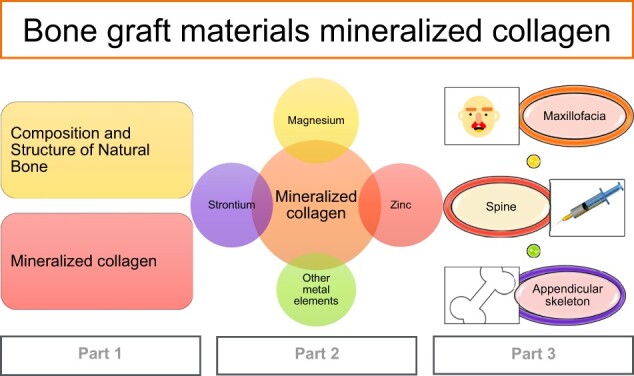

## Introduction

Musculoskeletal diseases affect more than one out of every two persons in the USA age 18 and over, and nearly three out of four age 65 and over. It was reported that musculoskeletal diseases account for more than 50% of disabling health conditions in adults [1]. Therefore, good biological materials that drive bone regeneration and remodeling have attracted much attention. An ideal bone defect filling material requires good biocompatibility and safety, osteogenic induction activity and an appropriate degradation rate, not only the degradation rate matches the bone healing rate but also the degradation products can be removed through a physiological excretion pathway or become a component of bone healing.

Mineralized collagen (MC) is composed of type I collagen and hydroxyapatite (HA) [2] and has the same chemical composition and hierarchical structure as natural bone [3]. MC scaffolds have high porosity and pore size similar to natural bone, and their good biocompatibility, degradability, low antigenicity and osteoconductivity make them of interest in the field of bone substitute materials. Inorganic metal ions play an important role in inducing bone tissue repair [4, 5] and have the advantages of lower cost, more stability and more effectiveness at low concentrations compared with protein growth factors [6]. Inorganic metal ions can rapidly diffuse across the cell membrane and regulate the activity of various cells. However, if distributed systemically, non-specific adverse effects may occur in the nervous, cardiovascular and endocrine systems [7, 8]. Therefore, osseointegration of therapeutic ions and biomaterial scaffolds can specifically target the damaged regions and has promising prospects.

This study reviewed the research on MC and its osseointegration with magnesium (Mg), strontium (Sr), zinc (Zn) and other metal elements in the repair of bone defects. This study provided a general introduction about collagen and HA was provided, summarized the biomineralization of collagen in natural bone and the influencing factors of MC preparation, and emphatically reviewed the advances in osseointegration of biomimetic mineralized collagen (BMC) and inorganic metal elements of natural bone for bone repair.

### Composition and structure of natural bone

**Figure 1.**
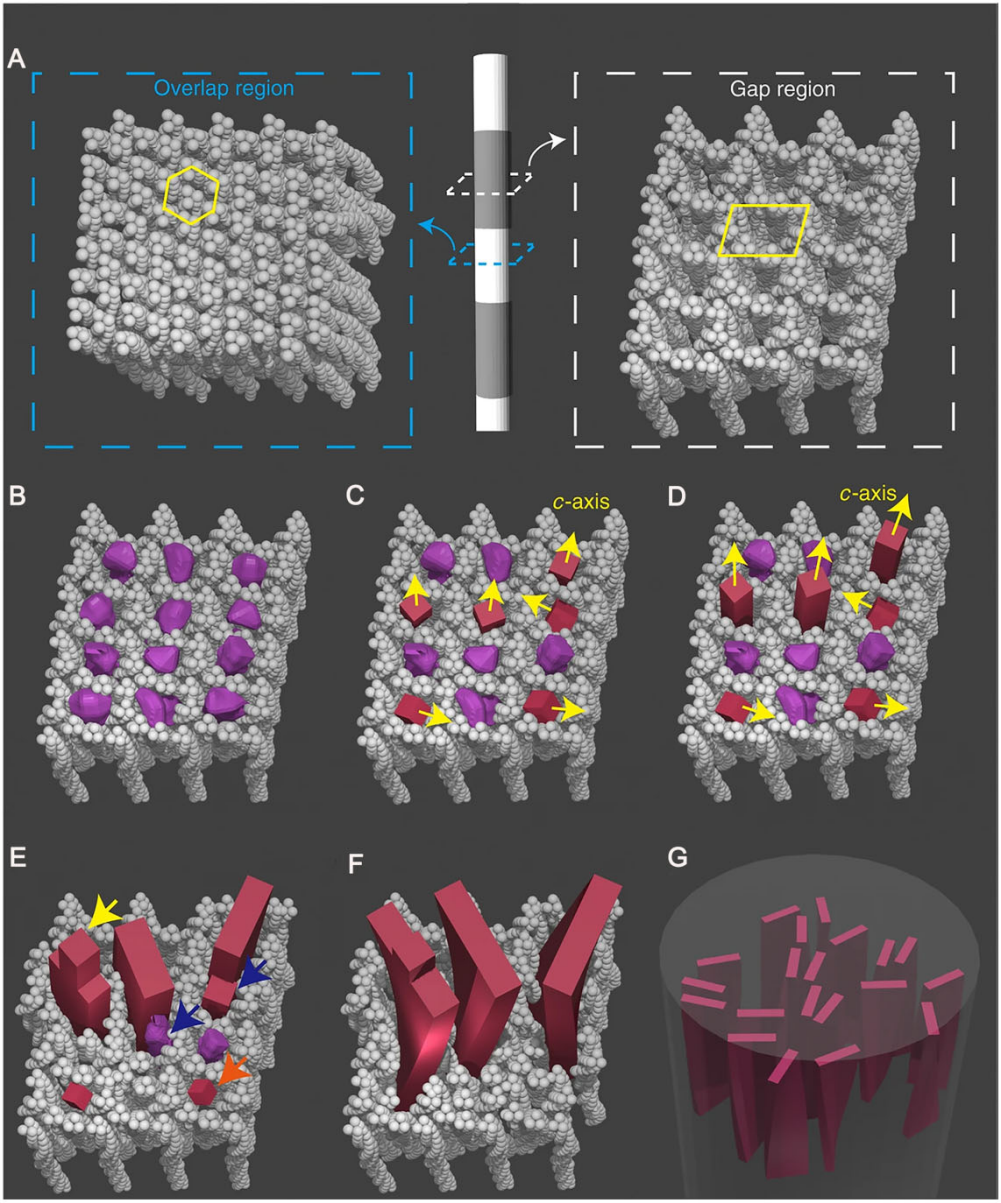
Schematic representation of the collagen mineralization model based on confinement. (**A**) The overlap region consists of tilted molecules that are densely packed in a quasi-hexagonal pattern, whereas the void region contains straight channels that allow HA nucleation (highlighted by the box). These channels initially fill the ACP phase (**B**), which forms the HA nuclei (**C**) through a transition from an amorphous to crystalline state. Nuclei with a rapidly growing *c*-axis parallel to the channel outperform nuclei with other orientations (**D**) and develop into platelets by pushing away collagen molecules, as shown in (**E**). Some platelets leave needle-like tips (highlighted by left arrow). Poorly oriented nuclei and ACPs are either consumed by Ostwald maturation (highlighted by right arrow) or fused to other platelets by adjusting the ionic arrangement (highlighted by arrows in the middle). Platelets grow further and begin to twist around their long axis, possibly due to changes in local collagen tissue (**F**). This results in a roughly uniaxial orientation of the twisted HA platelets with the *c*-axis parallel to the long axis of the fibril (**G**). Copyright 2020, The Authors.

The main components of native bone are inorganic minerals (about 65%) consisting of nanocrystals of carbonated HA; the organic phase (about 25%) includes extracellular matrix (collagen), non-collagenous proteins and cells; and water (about 10%). The basic building unit of natural bone is self-assembled collagen fibrils [9], type I collagen molecules self-assemble into collagen fibrils with a periodic 67 nm banded pattern and 40 nm gap zones between adjacent collagen molecules, and the collagen fibrils have nucleation sites for apatite crystal particles that guide the growth of mineral crystals [10, 11]. Eventually plate-shaped HA nanocrystals are modified in fibrils inside and outside the fiber [12, 13]. Intra-fibrillar mineralization is characterized by the distribution of HA within the interstitial zone of collagen fibers, with the c-axis of HA crystallites parallel to the long axis of collagen fibers [14]. Extra-fibrillar mineralization, on the other hand, is characterized by HA deposition on the surface of collagen fibrils or a gap between the two fibrils. In addition, the mechanism by which collagen guides the directional growth of HA crystals within fibrils is also a focus of research. For a long time, researchers have suggested that specific gap regions inside collagen fibrils contain rectangular (2D) channels, amino acid residues and charges in the channels that induce HA nucleation and epitaxial template crystal orientation. However, in recent years, it has been proposed that the orientation of HA crystals may be due to their restriction in collagen fibrils. Xu *et al.* [14] used X-ray diffraction (XRD) to observe the orientation of HA crystals in collagen fibrils and gap zones of unmineralized bone collagen in mineralized bone. The results showed the presence of elongated cylindrical nanopores in the unmineralized gap region, while the mineralized single collagen fibrils in human bone contained small stacks of a few crystals, and the mineral platelets only exhibited uniaxial orientation. The HA mineralization process was subsequently directly simulated in an *in vitro* model system. The results demonstrated that HA platelets were uniaxially oriented, which was achieved by confinement effects. Applying a similar simple strategy could help to design synthetic systems that simulate bone or promote its regeneration. Figure 1 shows a schematic representation of the collagen mineralization model based on confinement.

The excellent properties of bone result from the hierarchical arrangement of its microstructure and the collective contribution of its components [15]. The lamella is the most common structural component of the adult mammalian skeleton, and its basic structure consists of arrays of MC fibers arranged in form discrete layers. Raguin *et al.* [16] used the focused ion beam-scanning electron microscope to study the 3D structure of mineralized osteonal lamellar bone at nanometer resolution. Results identify two structural motifs of unidirectional and bidirectional arrays of MC fibers in porcine mineralized osteonal lamellar bone, the material containing predominantly bidirectionally aligned collagen fibrils is located between layers of predominantly unidirectionally aligned fibrils. In addition, it is commonly believed that in MC, collagen fibers are responsible for flexibility, while minreals are responsible for stiffness. Tavakol *et al.* developed a coarse-grained molecular dynamics framework to explore the mechanical properties of MC. It was found that in the large deformation state, the mineral phase of MC is responsible for limiting collagen sliding through high shear stress, which helps collagen molecules to withstand high tensile loads and provides an important contribution to the ultimate tensile strength of MC. Additionally, changes in the collagen network reduce the mechanical strength and elasticity of bone during aging [17]. Liu *et al.* [18] used atomic force microscopy, nanoindentation and Raman spectroscopy to investigate the response of collagen networks to aging and concomitant disuse. It was found that the D-periodic spacing and radial elastic modulus of a single MC and the mineral-to-matrix ratio of bone both increase with age, resulting in impaired microstructure and macromechanical properties of aged mouse bone. Besides, disuse mainly had a significant effect on the microstructure, mechanical properties and mineral-to-matrix ratio of the bones of aged mice, with little effect on young mice. In conclusion, the fine and well-ordered microstructure of bone confers excellent mechanical properties and biological function. It is worth studying and exploring how to design repair materials that are compatible with bone and improve osseointegration after implantation.

Type I collagen is the main structural protein of bone tissue, accounting for about 30% of bone dry weight and 90% of non-mineral content [19, 20]. Collagen can induce mineral deposition and has sites on its surface that promote osteoblast adsorption and mineral deposition [21]. Collagen is also effective in promoting mineralization and new bone formation in implanted artificial bone scaffolds. However, the disadvantages of pure collagen, such as poor mechanical properties, insufficient osteogenic stability and rapid degradation rate *in vivo*, affect its application in bone tissue engineering [22].

Most of the collagen in biomedicine comes from mammals, such as cattle and pigs, and such collagen may transmit infectious diseases, such as bovine spongiform encephalopathy and transmittable spongiform encephalopathies. Collagen derived from fish, jellyfish and other aquatic organisms have been extensively studied in recent years [23]. It has a wide range of sources, is easy to obtain, has good biocompatibility and has different amino acid sequences from humans, which can avoid or reduce immune responses. Aquatic collagen bioactive peptides also help absorb calcium and Zn [24]. For example, Xu *et al.* [25] isolated and derived collagen (DC) peptides from salmon, which can increase the contents of osteocalcin and alkaline phosphatase in the serum of rats and increase the density, dry weight and hardness of rat femurs. Aquatic collagen, however, has fewer proline and hydroxyproline residues and is less thermostable than bovine collagen [26]. Yao *et al.* [27] used acid dissolution and pepsin digestion to maintain the original composition and structure of type I collagen extracted from tilapia skin at low temperatures. In addition, Jeong *et al.* [28] demonstrated that duck’s feet-DC scaffolds could also be used as bone-graft materials. Thus, although different sources of collagen may still be inadequate, multiple sources of collagen still provide many options for implant preparation.

HA is the main inorganic component of bone and may bind various ions [29], such as Mg (Mg^2+^), potassium (K^+^), Zn (Zn^2+^) and manganese (Mn^2+^) [30]. Nano-hydroxyapatite (n-HA) is similar in size, crystallinity and chemical composition to HA in natural bone and is characterized by a rod-like or needle-like structure of 70–80 nm in length that binds tightly to the surface of recombinant collagen fibrils [31, 32]. In addition, n-HA adapts to the adhesion and growth of healthy osteoblasts and osteoclasts, and also promotes the value-added and differentiation of stem cells [33], which have excellent osteoconductive and osteoinductive activities in biological systems [15, 34]. Therefore, n-HA is a good raw material for the preparation of natural bone analogues.

### Physicochemical properties of MC

The combination of organic polymers and inorganic minerals is an effective way to improve mechanical properties [35]. This composite is synthesized similarly to the formation of bones, with organic templates driving the nucleation of the mineral phase, which is deposited directly in the organic matrix [36]. Collagen assembles into mineralized fibrils with n-HA, and subsequently, MC fibril bundles assemble in parallel to form interconnected porous structures similar to natural bone. MC with high porosity and cancellous bone with pore sizes of ∼100–300 um are structurally similar and provide interpenetrating channels that facilitate the ingrowth of cells, new bone and microvessels. Meanwhile, it has a strict hierarchical structure, reproducing the hierarchical structure of MC in natural bone. MC material has not only good biological properties but also has mechanical properties close to natural bone, and its compressive elastic modulus is close to the cortical bone in humans [37], while MC scaffold is 18 times stiffer than collagen scaffold (5.50 ± 1.70 versus 0.30 ± 0.09 kPa) [38]. In addition, MC can also promote cell adhesion and migration, angiogenesis and osteoblast differentiation, providing an appropriate cellular microenvironment for bone regeneration [39] and is a potential bone defect repair material.

The physicochemical process of biomineralization is performed in a physiological environment. The natural biomineralization process of natural bone is precisely regulated by biomacromolecules and some inducing factors, such as non-collagens (NCPs), and the final mineral phase, collagen, NCP, proteoglycan and water self-assemble into hierarchical structures with good mechanical properties [19]. In the preparation of natural bone analogue MC *in vitro*, mineralization pattern, mineralization time and the ratio of HA to collagen may affect its structure and function.

**Figure 2.**
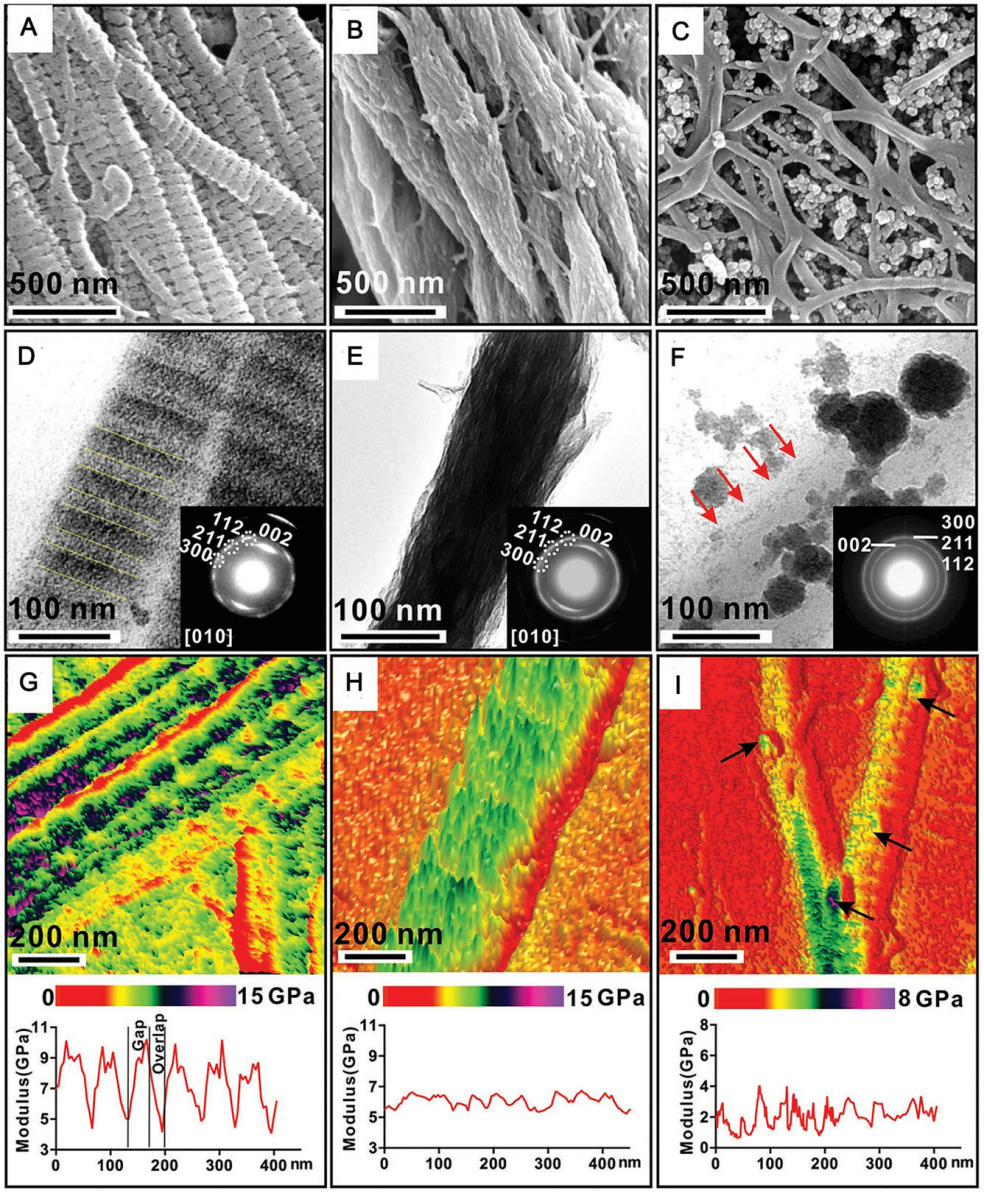
Nanomorphology and nanomechanical properties of MC. (**A–C**) SEM images of HIMC, NIMC and EMC. (**D–F**) Unstained TEM images of HIMC, NIMC and EMC. Nanopeptides are perpendicular to the repeated bands, and the *c*-axis of the crystallites is oriented preferentially along the collagen axis, as shown by the arcs of the 002 reflections in (D) (inset). No band pattern is found in (E). (F) Medium apatite crystals are deposited around the surface of a single nanofiber (arrows). (**G–I**) Corresponding AFM property maps and section analysis of Young’s modulus of (A) through (C). Periodic distribution of modulus with ≈67 nm is identified in (G) (upper), and section analysis shows a higher modulus in the gap zone of collagen and a lower modulus in the overlap zone of collagen in (G) (lower). The modulus of NIMC shows a uniform distribution in (H), an irregular distribution of modulus is observed in the EMC with extra-fibrillar apatite, and a higher modulus (arrows) is shown in (I). Copyright 2016, Wiley.

**Figure 3.**
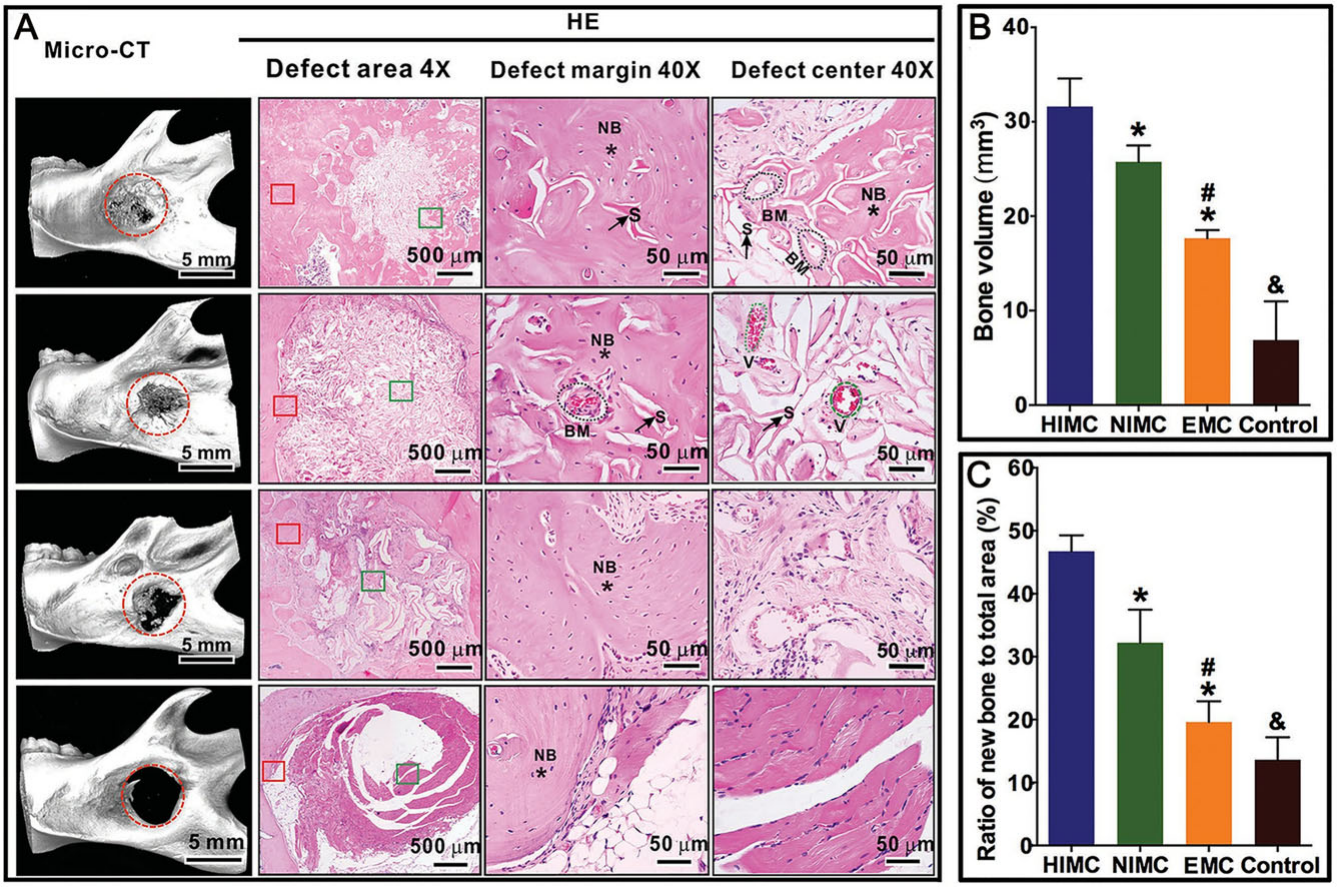
Experimental study of *in vivo* bone regeneration by different MC scaffolds. (**A**) Representative micro-CT and HE staining images of the mandibular defect areas 12 weeks after HIMC, NIMC and EMC scaffold implantation (left rectangle: defect margin; right rectangle: defect center). There are more new bone and marrow formations in the HIMC group. S: scaffold (arrows); NB: new bone (asterisks); V: vessel (circles); BM: bone marrow (circles). (**B**) The bone volume of bone defect areas from different groups was measured according to Micro-CT. (**C**) Semi-quantitative analysis of new bone on histological examination. *: *α* < 0.05 versus HIMC; #: *α* < 0.05 versus NIMC; &: *α* < 0.05 versus all of the other groups. Copyright 2016, Wiley.

The microstructure, mechanical properties and biocompatibility of MC scaffolds prepared by different mineralization methods have some differences. In vertebrate bones, extra-fibrillar mineralization is manifested by the random accumulation of HA microcrystals on the surface of collagen fibers or in the interstices between two fibers, which provides the load-bearing properties of the bone but not the same surface appearance and nanostructure as the bone extracellular matrix [40, 41]. Intra-fibrillar mineralization is shown by the distribution of HA within the interstitial zone of collagen fibers, with the c-axis of HA crystallites parallel to the long axis of collagen fibers. Natural bone is a complex system of synergistic intra- and extra-fibrillar MC fibers, with a volume ratio of extra-fibrillar mineralized collagen (EMC) to intra-fibrillar MC of ∼3:1. Ideal natural bone analogue would have both extra-fibrillar and intra-fibrillar mineralization with similar volume ratios, but to date it has been difficult to achieve this high level of biomimetics. So many studies have compared the physicochemical properties and biocompatibility of intra-fibrillar minrealized collagen with EMC in different hierarchical structures. Liu *et al.* [42] used polyacrylic acid (PAA), an analogue of NCPs with different molecular weights, to regulate nucleation and growth of n-HA in collagen fibrils to prepare layered intra-fibrillar mineralized collagen (HIMC), non-layered intra-fibrillar mineralized collagen (NIMC) and EMC. The hierarchical structure of HIMC provides excellent strength similar to natural bone, as shown in Fig. 2 for the nanomorphology and mechanical properties of each scaffold. *In vitro* and *in vivo* experiments have demonstrated that HIMC scaffolds can provide a better microenvironment to promote osteogenic differentiation of stem cells and promote bone regeneration. Figure 3 shows the bone regeneration of each scaffold in SD-Dawley rats. The results demonstrate that it is possible to prepare a bone tissue engineering scaffold HIMC that mimics chemical and physical complexity of natural bone by bottom-up mineralization, which can provide suitable mechanical properties and optimized microenvironment to regulate osteogenesis, as well as nanostructure to improve bone regeneration. Similarly, Wang *et al.* [43] compared the mechanical properties and biological characteristics of 3D BMC scaffold with intra-fibrillar mineralization, traditional mineralized collagen (TMC) scaffold with extra-fibrillar mineralization and non-MC scaffold. Results showed that BMC scaffold had a much better modulus of elasticity (41.64 ± 3.72) than TMC scaffold (24.64 ± 2.33). Besides, BMC scaffold had better biocompatibility and the ability to induce regeneration of bone defects than other scaffolds. Therefore, MC scaffold with layered interlacing and HA microcrystals selectively deposited the interstitial zone of collagen fibers is currently the optimal solution to mimic natural bone.

The preparation of intra-fiber mineralization is also influenced by a number of factors. Nowadays, intra-fibrillar MC scaffolds are usually prepared by the polymer-induced liquid precursor (PILP) method, NCPs analogues stabilize amorphous calcium phosphate (ACP) and facilitate its penetration into the interstitial zone of collagen fibers [44]. For example, Wang *et al.* [45] first prepared an acidic collagen solution by dissolving type I collagen in dilute hydrochloric acid. Then, added calcium dichloride solution and monosodium phosphate solution to the acidic collagen solution dropwise while stirring. After adequate mixing, the PH value was adjusted to 7.4. The MC deposition was gradually formed. In addition, currently, PAA is the most widely used NCPs analogues, which can provide an abundant negative charge, mimic the structure and function of different NCPs and have good biocompatibility [46]. NCPs analogues also include carboxymethyl chitosan [47], poly-L-aspartic acid [48, 49], osteopontin [49], alginate [50], amorphous calcium fluoride [51], etc. In addition, Gibbs–Donnan equilibrium indicates that both short-range electrostatic forces driven by charge distribution and long-range osmosis driven by osmotic pressure between the extra-fibrillar and intra-fibrillar water compartments may influence the mineralization process within the fibril. Capillarity may also be involved in intra-fibrillar mineralization, and capillary forces are expected to provide rapid and long-range transport of fluid precursors. In addition, effective nanoscale prestress can enhance the biomineralization strength of materials. Ping *et al.* [52] simulated *in vivo* collagen fibril mineralization by PILP using collagen substrates, such as SrCO_3_, SrWO_4_, SrSO_4_, Ca_10_ (PO_4_)_6_(OH)_2_, CaF_2_ and CaCO_3_, and monitored the mineralization process and internal stress formation of the collagen matrix by X-ray scattering, optical microscopy and so on. The results showed that the stresses in the collagen fibers are transferred to the embedded minerals, causing the lattice to be strongly compressed, thus the mechanical properties of mixed materials can be improved by internal stresses. Therefore, if the natural bone mineralization process can be simulated to the greatest extent during collagen mineralization *in vitro*, better implants may be obtained by providing collagen substrates, extra-fibrillar and intra-fibrillar electrostatic forces and osmotic pressure differences while preparing MC by the PILP method.

The ratio of n-HA and type I collagen also impacts the structure and function of the scaffold. Ou *et al.* [53] performed *in vitro* and *in vivo* studies of composite scaffolds with HA/Col mass ratios of 9:1, 7:3, 5:5 and 3:7. The results showed that HA/Col scaffolds with a 7:3 mass ratio had better cell adhesion and osteogenic induction ability. Yao *et al.* [27] compared T-col/HA scaffolds with tilapia skin collagen and HA mass ratios of 1:4 and 1:9 and found that mechanical strength was increased 17-fold and 32-fold, respectively, compared with pure collagen scaffolds. Additionally, all scaffolds showed some osteogenic activity, while the 1:4 T-col/HA scaffolds had the strongest ability to promote cell proliferation and osteogenesis *in vitro* and *in vivo*. Therefore, when the ratio of n-HA and collagen I is close to the composition of native bone, it tends to have a better ability to promote bone regeneration. Sun *et al.* [54] investigated scaffolds with 3:7 and 5:5 volume ratios of n-HA and Col I and n-HA particles in scaffolds with 3:7 volume ratios were evenly distributed on the pore walls formed by collagen matrices to form stable and uniform porous structures. The scaffold also significantly promoted the expression of osteogenesis-related genes Runx-2, BMP-2 and OCN. This provides a new idea that the composition ratio of HA/Col affects the structure and function of the scaffold.

## Osseointegration of MC and metal elements

Natural bone is mainly composed of n-HA and collagen fibers, and trace elements, such as Mg, Sr and Zn, also play an important role in bone formation [55]. The main inorganic component of MC is n-HA [Ca_10_(PO_4_)_6_(OH)_2_], whose crystal structure ascribes to the hexagonal crystal system, giving it flexibility and stability in structure and composition. This crystal structure makes it allow the plasma substitution of many ions, such as calcium ions, and phosphate [56]. In the HA lattice, (PO_4_)^3−^ tetrahedra are linked together by Ca^2+^ bridges. The relatively large space between the (PO_4_)^3−^ groups allows the accommodation of foreign atoms that are quite different from the Ca2 + diameter. (OH)^−^ ions align along the 6-fold axis of the lattice bounded by Ca^2+^ and (PO_4_)^3−^ columns, forming the so-called ‘apatite channel’ [33]. Metal cations, such as Ag^+^, Na^+^, Mn^2+^, Mg^2+^ and Sr^2+^, replace Ca (I) (surrounded by phosphate) and Ca (II) (surrounded by hydroxyl) [56] in HA lattices, (CO_3_)^2−^ anion replaces the (OH)^−^ and (PO_4_)^3−^ sites. The osseointegration of MC with natural bone inorganic metal elements is a promising topic of current research.

### Magnesium

Mg can modulate related hormones and factors to affect bone metabolism [57]. Mg deficiency leads to decreased osteoblast activity, decreased levels of markers of osteoblast function, and even osteoporosis [58]. The osseointegration of Mg with MC has good synergistic effects in bone defect repair. Mg stabilizes ACP and inhibits the growth of HA crystals along the c-axis, contributing to the preparation of composite scaffolds with uniform mineral distribution and surface roughness similar to natural bone [36, 59]. In addition, MC scaffolds loaded with Mg ions can activate the PI3K/AKT/GSK3b/b-catenin signaling pathway to significantly enhance osteogenic mineralization of cells. The content of Mg in the composite and the sustained release of Mg too high and too low will affect its bone repair effect. Sun *et al.* [54] found that MC3T3-E1 cells significantly promoted Runx-2, OCN and BMP-2 gene expression when cultured in a medium containing 12 mmol/l Mg^2+^. Therefore, when the Mg content in the implantation site of the composite is close to that in 12 mmol/ Mg^2+^ medium, it can significantly promote the osteogenic properties of the scaffold. It has also been shown that Mg exhibits a very high rate of cell adhesion and bone-contributing ability at molar ratios of no more than 10% in HA [33, 60].

**Figure 4.**
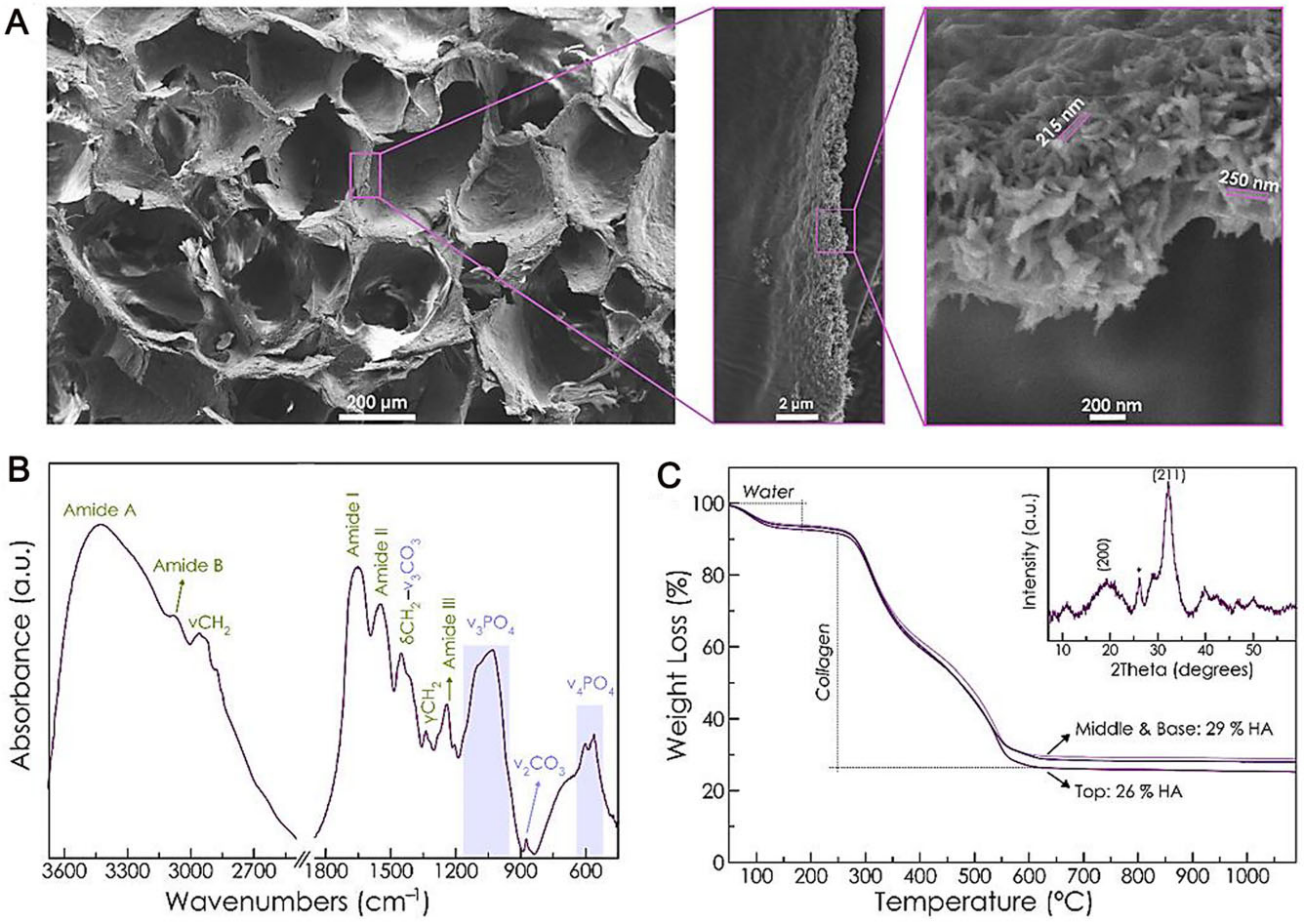
Scanning electron microscopy images of RCP.MgAp. (**A**) Scanning electron microscopy images of cross-section of RCP.MgAp scaffold. A high magnification image of the walls showed apatite (Ap) into the organic matrix. (**B**) Fourier-transform infrared spectroscopy of the RCP.MgAp scaffold. (**C**) Thermogravimetric curves of the top, middle and base of the scaffold. Inset shows the XRD pattern of the sample with the characteristic reflection of HA (ASTM card file No 09-432). Copyright 2021, MDPI.

Because natural collagen is difficult to process and purify, varies from batch to batch, and may spread diseases, recombinant collagen that can introduce specific amino acid sequences has received a lot of attention [61]. Rodríguez *et al.* [59] investigated biomimetic mineralization of recombinant collagen peptide (RCP) containing the arginine–glycine–aspartate sequence in the presence of Mg, and obtained 3D porous scaffolds with pore sizes of 50–600 µm and porosities of 90 ± 0.4% by freeze-drying, needle nanoparticles nucleated and evenly distributed into the organic matrix (RCP.MgAp), which could promote the proliferation of bone marrow mesenchymal stem cells, chemical signaling of osteogenesis and up-regulation of osteogenic genes. Therefore, Mg with collagen peptides for biomineralization is a promising solution for the repair of bone defects. Figure 4 shows scanning electron microscopy images of RCP.MgAp.

**Figure 5.**
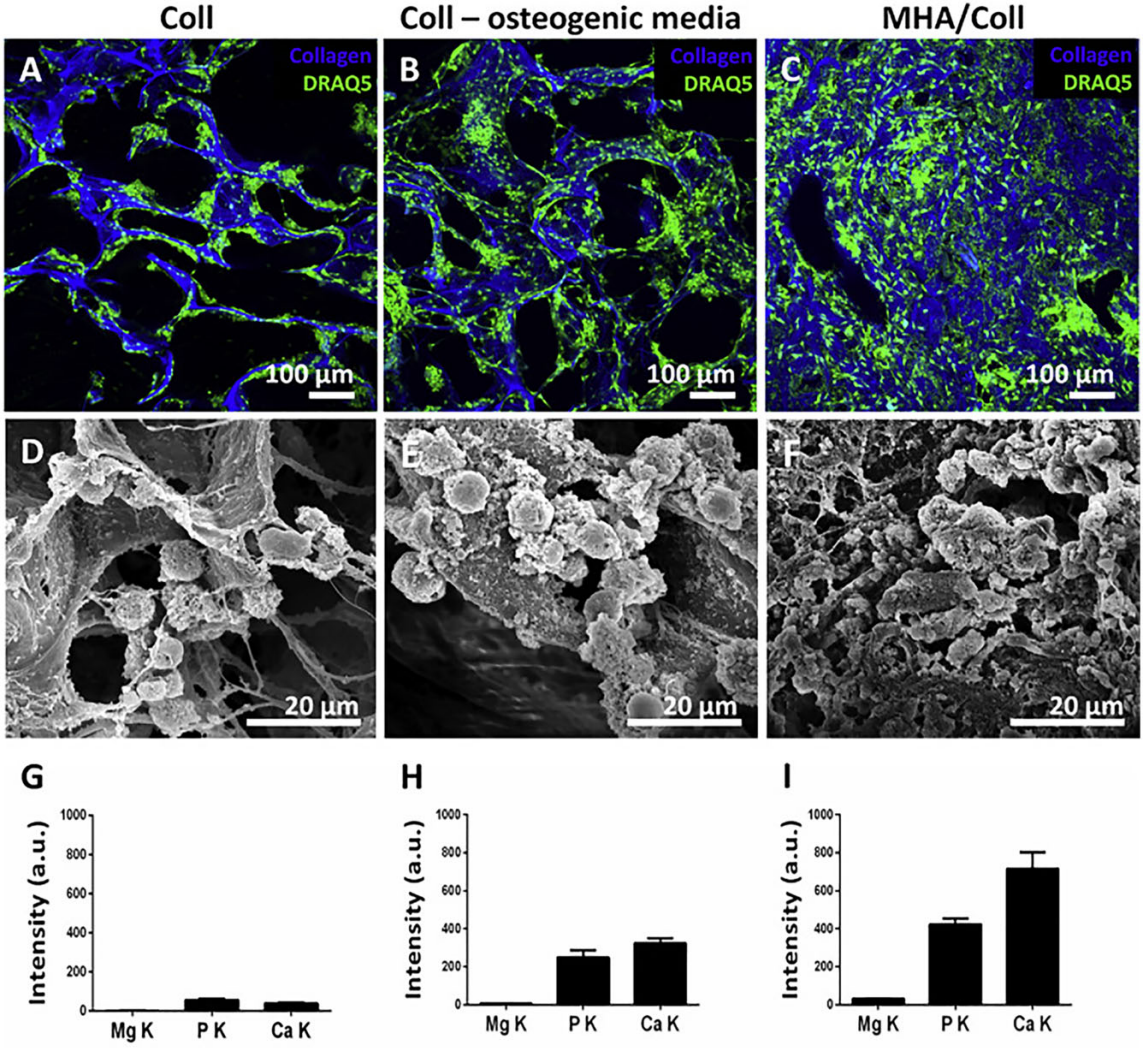
Microscopic observation of the morphology of HBM-MSCs. The morphology of HBM-MSCs in COL and osteogenic medium after 3 weeks of culture was observed by confocal laser scanning microscopy (**A–C**) and scanning electron microscopy (**D–F**). The relative content (intensity) of Mg, phosphorus and calcium in the scaffolds of three experimental groups was quantified by energy dispersive analysis. Data were normalized to cell-free Coll and MHA/Coll scaffolds as baselines. Copyright 2020, The Authors.

The Mg-doped HA/type I collagen (MHA/Coll) scaffold prepared by Minardi *et al.* [36] had interconnected pores with a porosity of about 70%, the scaffold structure was dense and not loose, Mg-doped HA (MHA) was uniformly nucleated along the collagen bundle, collagen fibers were completely mineralized with amorphous mineral phases, and the significant mineralization most of *de novo* mineralization was found. When cells were seeded on the scaffolds, MSCs were found to be arranged in large clusters in the pores, as shown in Fig. 5. Also, key marker genes and Mg transporter 1 were significantly up-regulated at each stage of osteogenic differentiation and maturation, and the MHA/Coll scaffold enhanced calcium deposition and osteogenic differentiation of MSCs [62]. After 6 weeks of implantation, extensive trabecular bone-like bone and corticoid bone tissue was formed. *In vivo* studies have found that the MHA/Coll scaffold is effective in recruiting progenitors and osteocytes and contributes to the assembly of the hematopoietic ecosystem. Sun *et al.* [54] demonstrated that MC scaffolds loaded with Mg–Sr ions could activate PI3K/AKT/GSK3b/b-catenin signaling pathway to promote osteogenic differentiation of cells. So the MHA/Coll scaffold has good osteoinductive potential and provides a direction for clinical bone repair.

However, Roffi *et al.* [63] prepared [MHA/(Col+chit)] composite scaffolds by biomimetic mineralization processes to align MHA nanoparticles in Col+chit blends. The results of experiments performed on sheep and rabbit models showed no osseointegration, even a clear separation between bone and scaffold was detected, and histological examination revealed that the bone defect area was always identifiable. Both models demonstrate that MHA/(Col + chit) scaffolds lack regenerative potential at both skeletal and cartilage levels. While it may be that the high degree of crosslinking reduces the availability of active charged sites on the chitosan surface related to water adsorption, the use of different chitosan formulations may lead to different results. Interactions between different materials may also result in composite scaffolds failing to provide bone and cartilage regeneration *in vivo*. Therefore, there is still much room for the development of HA/Col and Mg osseointegration.

**Figure 6.**
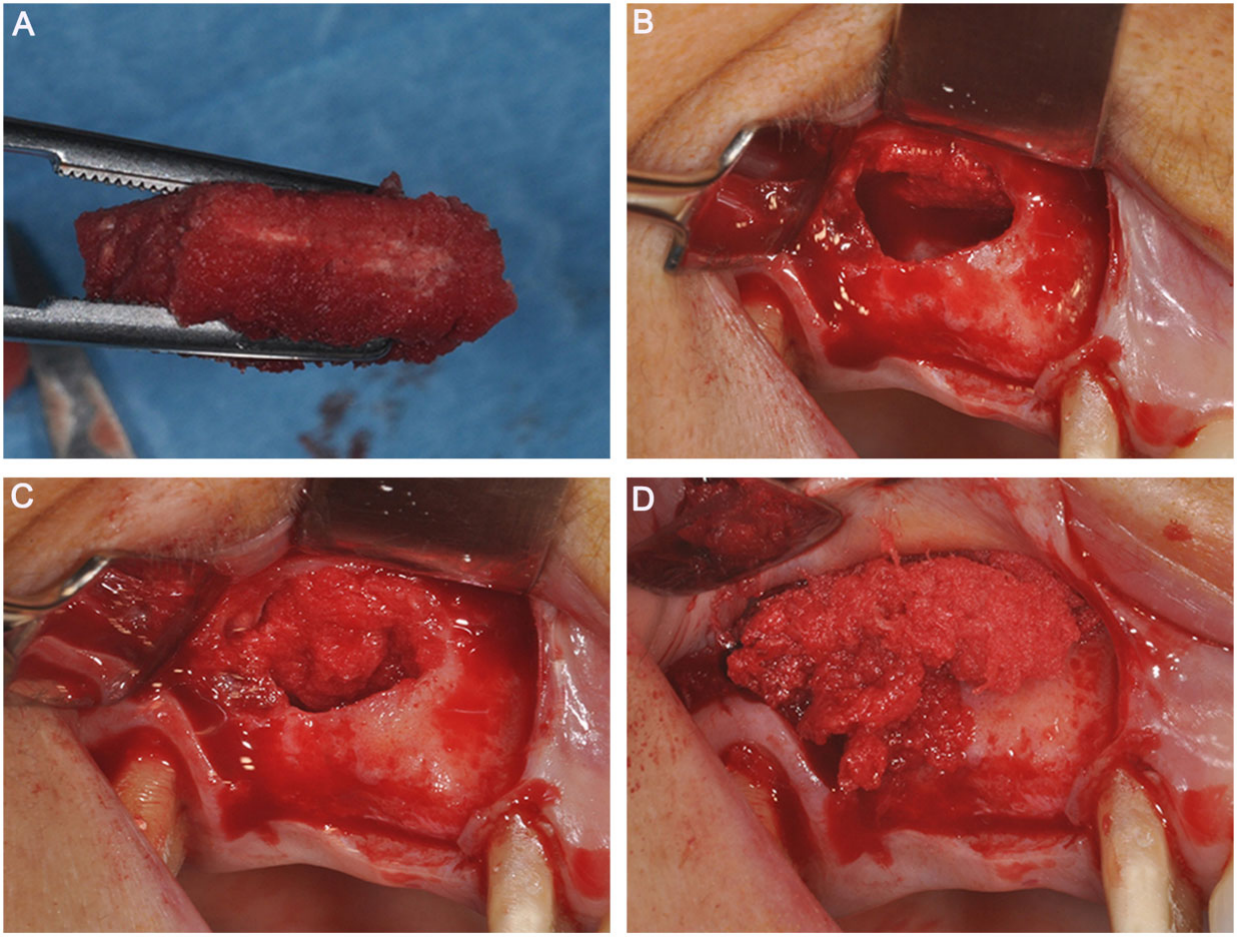
Regen Oss is used in sinus augmentation. (**A**) Blood molecules and cells that promote bone formation are rapidly absorbed during graft placement. (**B**) Expose the lateral wall of the maxillary sinus and incise a bone window. (**C**) Commercial Mg HA/collagen-based scaffolds were filled in the maxillary sinus. (**D**) During sinus filling, the scaffold easily adapted to the dimension and shape of the sinus, saving time and improving sinus filling. Copyright 2017, Scarano.

Commercial Mg HA/equine collagen I scaffolds (MHA nanocrystal/collagen scaffolds) (Regen Oss) have also been used clinically. Regen Oss is made of nanoscale (10–20 nm) biomimetic MHA crystals (70%) and type I collagen fibers (30%), which are evenly distributed in biopolymerized collagen. Taschieri *et al.* [64] investigated the use of Regen Oss in alveolar socket preservation, where scaffolds were almost completely replaced by newly formed bone tissue after 6 months. Scarano *et al.* [65] used Regen Oss for sinus augmentation, as shown in Fig. 6. The results showed that the scaffold could easily adapt to the size and shape of the paranasal sinuses and improve sinus filling. Six months after surgery, new bone formation was observed at the grafted site with no material remaining, and tomography showed cortication of the buccal window of the paranasal sinuses, confirming good bone-graft healing. Therefore, MHA/Col scaffold has the advantages of easy operation, absorption, promoting bone regeneration, etc., making it an option for clinical bone defect repair.

### Zinc

**Figure 7.**
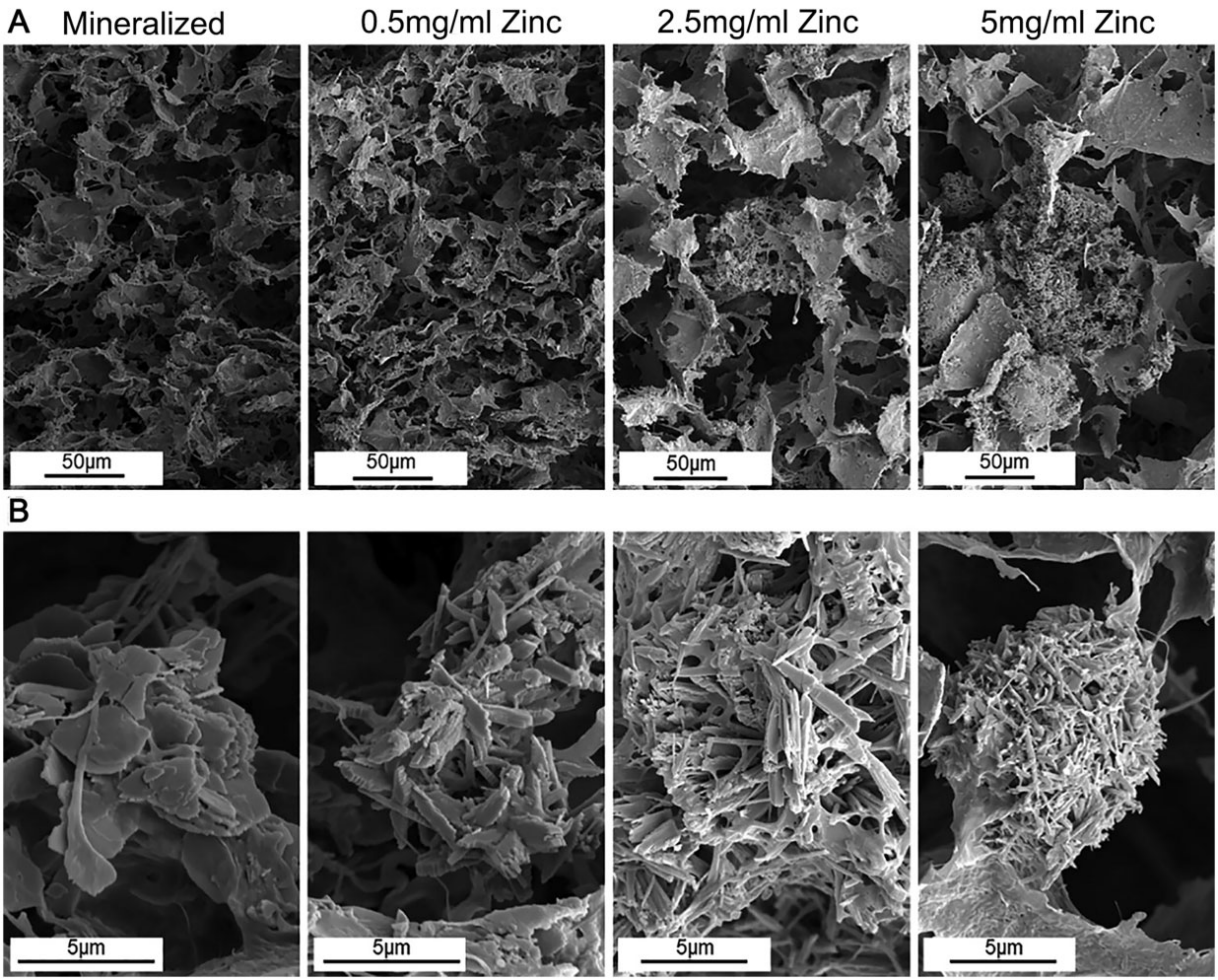
Scanning electron microscopy images of mineralized Zn scaffolds. (**A**) There was no significant change in the pore structure of different scaffolds, but there was a significant change in crystal aggregates in the 2.5 and 5 mg/ml groups. (**B**) The addition of Zn altered the brushite structure within the scaffold, changing from plate-like crystals to elongated needle-like structures. Copyright 2019, Acta Materialia Inc.

Zn is essential for bone formation and mineralization and is found predominantly in skeletal tissue. Zn activates MAPK kinases and stimulates the proliferation of osteoblasts in bone tissue [66]. Zn deficiency can lead to bone growth retardation, mineralization abnormalities, osteoporosis and even dwarfism. Osseointegration of Zn with MC can increase mineral deposition and modify the microstructure of minerals, with little effect on their pore and mineral phase composition [67]. In addition, Zn complex MC also promotes BMSC migration and differentiation and angiogenesis by activating the p38 MAPK pathway in monocytes [68]. Numerous studies have shown that elemental Zn significantly improves the mechanical properties, biocompatibility and osteogenic induction activity of MC scaffolds, contributing to their use in the repair of bone defects. For example, Aleczandria *et al.* [67] prepared MC–glycosaminoglycan–Zn scaffolds containing Zn ions at 0, 0.5, 2.5 and 5 mg/ml Zn ions. Scanning electron microscopy (Fig. 7) showed that the addition of Zn changed the brushite structure in the scaffold from a plate-like crystal to an elongated needle-like structure, and the pore structure of the scaffold did not change significantly, while the crystal aggregates in the 2.5 and 5 mg/ml groups changed significantly, and the elastic modulus of the scaffold also increased significantly. Zn-modified scaffolds can also improve the cellular metabolic activity of primary pig-derived adipose stem cells.

**Figure 8.**
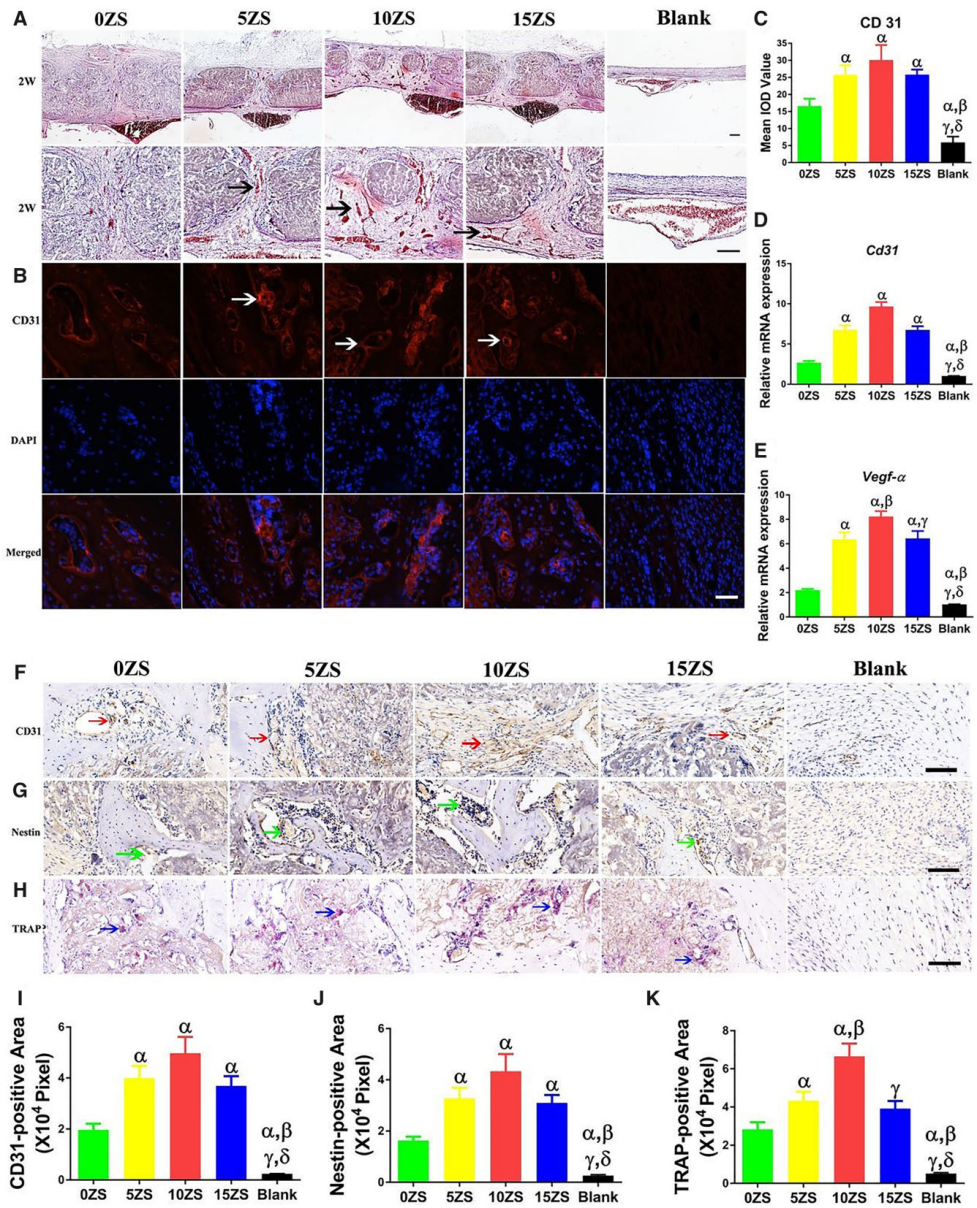
Assessment of angiogenesis in rat critical skull defects implanted with ZS/HA/col scaffolds containing different proportions of zinc silicate. No scaffolds were implanted in the blank group. (**A**) H&E staining of decalcified bone specimens at 2 weeks after implantation (arrows: vessels. Scale bar: 200 μm). (**B**) Immunofluorescence staining for CD31 (arrows) and DAPI (blue) 2 weeks after implantation (scale bar: 200 μm]. IOD quantitation of CD31 (**C**). Statistical comparisons of the relative CD31 (**D**) and VEGFR-α (**E**) gene expression at 2 weeks after implantation. Immunohistochemical staining for CD31 (3**F**, arrow), Nestin (3**G**, arrow) and TRAP (3**H**, arrow) (scale: 200 μm) was performed 2 weeks after implantation of ZS/HA/COL scaffolds containing different proportions of zinc silicate into rat cranial defects. ^α^*P* < 0.05 versus 0 ZS/HA/Col scaffold; ^β^*P* < 0.05 versus 5 ZS/HA/Col scaffold; ^γ^*P* < 0.05 versus 10 ZS/HA/Col scaffold; ^δ^*P* < 0.05 versus 15 ZS/HA/Col scaffold. Copyright 2020, American Chemical Society.

Song *et al.* [68] prepared Zn silicate/n-HA/type I collagen (ZS/HA/Col) scaffolds containing 0, 5, 10 and 15 wt% zinc silicate, and physical encapsulation of zinc silicate in collagen allowed the sustained release of Zn and silicon ions *in vivo*. The results showed that zinc silicate had no effect on porosity and pore size, and a higher proportion of zinc silicate could significantly increase the compressive elastic coefficient of the scaffold. The ZS/HA/Col scaffold also promoted the differentiation of monocytes into TRAP^+^ cells and activated the p38 MAPK pathway in monocytes to increase the recruitment of BMSCs and endothelial cells and promote osteogenesis and angiogenesis. As shown in Fig. 8, the assessment of angiogenesis in critical skull defects in rats implanted with ZS/HA/Col scaffolds with different ratios of zinc silicate. Therefore, osseointegration of Zn with MC can be developed in different ways and has great potential for application in the repair of bone defects.

However, the structure and morphology of chit/aga/HA-Mg scaffolds and chit/aga/HA-Zn scaffolds prepared by Paulina *et al.* [69] were not significantly changed compared with scaffolds without Mg and Zn. The results showed that chit/aga/HA-Mg scaffold could promote integrin-mediated cell adhesion, spreading and proliferation, but chit/aga/HA-Zn could not promote cell proliferation. Scaffold neither promoted the synthesis of osteogenic markers nor promoted a significant decrease in the mineralization activity of stem cells. This result, which is different from the general perception, suggests that there are many factors, such as mechanical and physicochemical properties, the degree of substitution of metal materials and the release rate of ions that may affect the effect of implanted materials.

### Strontium

Sr is a strong bone-seeking trace element. Sr increases alkaline phosphatase activity and promotes matrix mineralization and osteogenic differentiation [6]. Sr^2+^ ions can affect Wnt/β-catenin and RAS/MAPK signaling pathways to promote osteogenesis. Sr has been widely used in the treatment of osteoporosis and other diseases. For example, Meunier *et al.* [70] demonstrated that daily administration of 2 g Sr ranelate to postmenopausal women increased bone mineral density and significantly reduced fracture risk. Osseointegration of elemental Sr with MC reduces nucleation barriers, regulates nucleation sites and promotes more homogeneous mineralization within fibrous regions [55]. The MC scaffold loaded with Sr ions also activates the PI3K/AKT/GSK3b/b-catenin signaling pathway and the MAPK pathway significantly enhancing the expression of osteogenic genes and proteins at the cellular level [54, 71]. The composite scaffold can accelerate the reconstruction of bone tissue and promotes the formation of osseointegration between the implant and the new bone. In addition, local controlled release of Sr ions is the focus of research. Sun *et al.* [54] compared MC3T3-E1 cells cultured in medium containing Sr^2+^ at 0, 3, 6 and 12 mmol/l. The results showed that 3 mmol/l could significantly promote the expression of osteogenic-related genes, such as Runx-2, BMP-2 and OCN. Therefore, when the content of implants in the implantation site is close to 3 mmol/l, it can well affect its osteogenic properties. Quade *et al.* [72] prepared 25%, 50%, 75% and 100% Sr-modified MC scaffolds with 25% scaffolds containing 0.8 mmol g^−1^ Sr and 100% scaffolds containing 4.9 mmolg^−1^ Sr. As the amount of Sr substituted in the mineral phase increases, the degree of mineralization of collagen increases, porosity decreases from 84% (Sr0) to 80% (Sr100), and its compressive strength remains unaffected. Therefore, Sr and MC composite scaffolds have potential applications for the treatment of defects in damaged bone throughout the body.

Liu *et al.* [55] found that most of the mineralized crystals in the gelatin-SrHA samples were located in the fibril zone and deposited uniformly along the fibril compared to the gelatin-HA samples. MC3T3-E1 cells showed better attachment and spreading ability on gelatin-SrHA samples. Therefore, Sr ions can regulate the nucleation location and mineralization behavior of gelatin and play a significant role in maintaining cell morphology and internal structural order, cell migration and intracellular signal transduction. Chen *et al.* [71] first prepared Sr−graphene oxide (GO) nanocomposites, which were then amidated to form Sr–GO–Col scaffolds. Sr–GO–Col has a highly porous and interconnected structure, with a 3.1-fold increase in elastic modulus and significantly improved hydrophilicity and water retention compared with unmodified collagen scaffolds, which can maintain a moderate concentration of Sr ion release for a long time and contribute to cell adhesion. Sr–GO–Col scaffolds also activated the MAPK signaling pathway to significantly increase osteogenic gene and protein expression levels in human adipose-derived stem cells. Zhang *et al.* [73] developed a biomimetic composite composed of Sr/copper doped HA (Sr/Cu-doped 1D HA) and poly (D, L-lactide), which has comparable compressive strength, tensile strength and Young’s modulus to cortical bone. The continuous release of incorporated Sr and copper ions not only promoted the attachment, proliferation and differentiation of MSCs, but also induced the orientation of hMSCs and the formation of an anisotropic collagen fibril matrix. Therefore, osseointegration of Sr with MC has great potential for application in bone defect repair.

**Figure 9.**
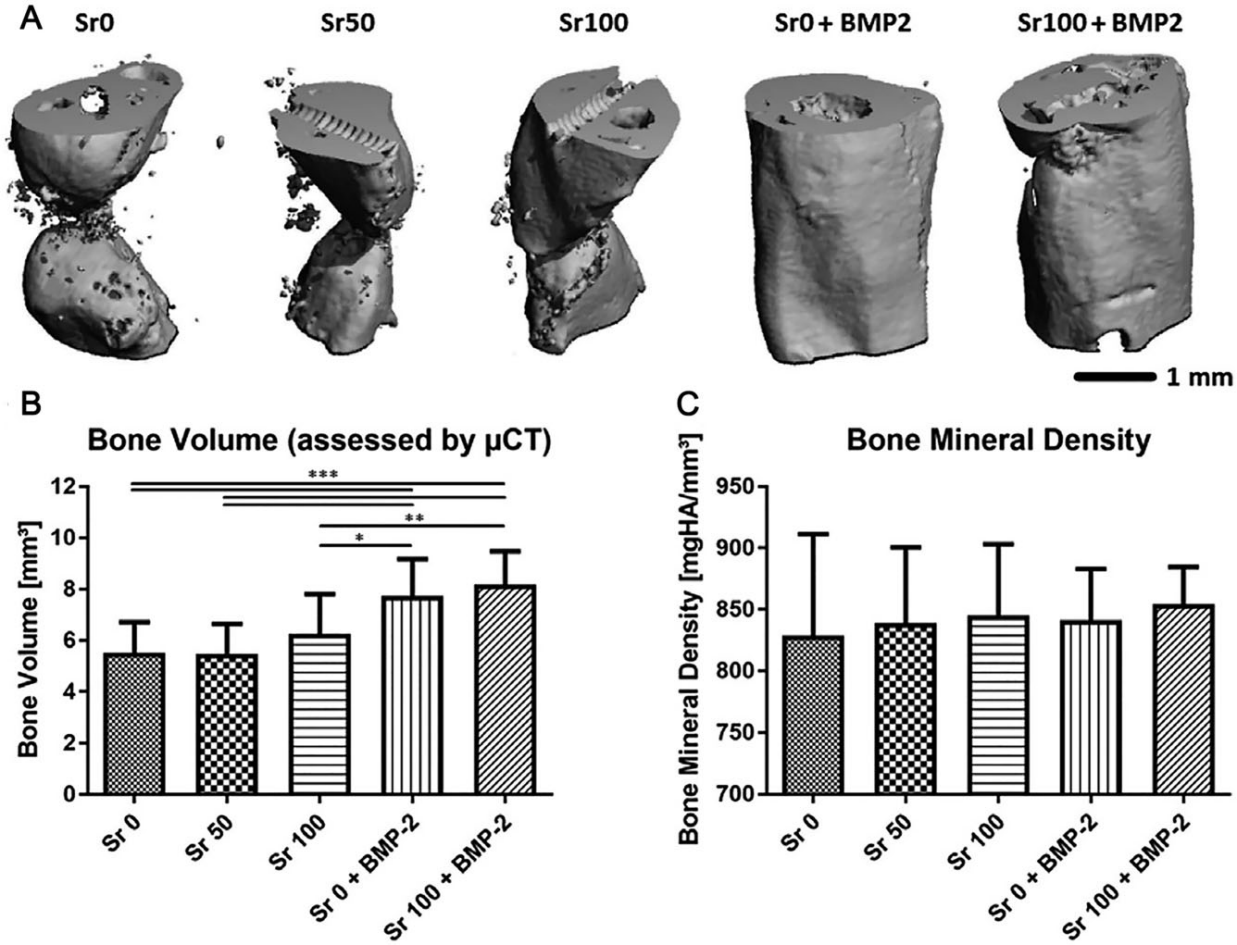
New bone formation in the femoral defect region of mice filled with MC scaffolds modified with Sr (Sr0, Sr50 and Sr100) and BMP-2 (Sr0 + BMP-2, Sr100 + BMP-2). (**A**) High-resolution micro-CT evaluation of bone volume in the defect area. (**B**) Volume. (**C**) Mineral density of newly formed bone. Mean ± SD, *P* < 0.05, *P < 0.01, *P* < 0.001. Copyright 2019, Wiley.

Quade *et al.* [74] investigated the bone regenerative capacity of Sr-heparinized MC scaffolds combined with BMP-2 in mouse bone defect models. The results showed that the scaffold could promote the proliferation and osteogenic differentiation of human MSCs. MC scaffolds with complete replacement of calcium ions by Sr ions combined with BMP-2 healed the bone defects best, as shown in Fig. 9. Therefore, combining BMP-2 with Sr-modified MC scaffolds is a promising strategy to promote bone regeneration.

### Other inorganic metal elements

Iron (Fe) plays a crucial role in bone metabolism [75], and Fe deficiency leads to abnormalities in collagen synthesis, maturation and metabolism [76]. Magnetic nanoparticles, such as FeHA and Fe_3_O_4_, can be complexed with MC as scaffolds, coatings [77] and hybrid microspheres [78, 79], which can impart magnetic properties to mineralized colloidal materials, deliver cellular command signals in response to different environmental stimuli and positively influence the expression of osteogenic markers. For example, Anna *et al.* [80] prepared a bionic hybrid scaffold with superparamagnetic properties by dropping a collagen suspension into an alkaline suspension containing Fe^2+^/Fe^3+^ ions, the FeHA/Coll scaffold prepared at higher temperatures (50°C) was found to have not only good stability but also good biocompatibility, supporting the adhesion and proliferation of MG63 human osteoblast-like cells. In addition, the composite material prepared by combining Fe and MC can also influence the mechanical stimulation of the implant surface to promote osteogenic differentiation. For example, Junjun *et al.* [77] electrochemically applied iron oxide nanoparticle/MC (IOP-MC) coatings to titanium alloy substrates. It was found that the IOP-MC coating with an IOP to MC mass ratio of 0.67 was able to achieve mechanical stimulation of the implant surface under a static magnetic field and showed very significant osteogenic differentiation activity. Therefore, osseointegration of Fe with MC enhances osteoblast adhesion, osteogenic differentiation of mesenchymal stem cells and magnetic fields for bone tissue regeneration, giving it great potential for applications in bone defect repair. In addition, its combination of biomimicry and the ability to remotely activate and release bioactive molecules along a specific spatio-temporal distribution provides directions for both bone fillers and drug delivery systems in bone tissue engineering.

Manganese (Mn) is one of the most important trace elements in bone [81]. Mn deficiency leads to delayed osteogenesis, bone deformation, inhibition of growth and even bone resorption. Westhauser *et al.* [82] found that Mn could improve the survival rate of MSCs and promote osteogenesis within certain limits. Osseointegration of Mn with MC does not significantly interfere with the mineralization process within the collagen fibers, and the composite scaffold maintains a porous, layered laminar structure that significantly promotes the osteogenic differentiation of cells. Yu *et al.* [4] prepared 10 mol% Fe^2+^ and 10 mol% Mn^2+^, 5 mol% Fe^2+^ + 5 mol% Mn^2+^ replaces the 10 mol% Ca^2+^ Col-FeHA-lamellar, Col-MnHA-lamellar and Col-FeMnHA-lamellar scaffolds. It was found that the scaffolds showed lamellar porous structures ranging from macroscopic to microscopic. All scaffolds promoted the expression of osteoblast proliferation and adhesion, osteogenic differentiation-related genes, such as BSP, DMP1, Col-1 and so on. Scaffolds loaded with both Fe and Mn had the strongest ability to contribute to the bone. After adding Fe and Mn, *in vivo* experiments also confirmed that the bone regeneration ability was significantly enhanced. Therefore, MC combined with Fe and Mn has the potential to be an option for bone defect repair.

Terbium (Tb) showed special luminescent properties at 544 nm, making it one of the rare earth probes for luminescence. Tb can up-regulate the expression of adhesion molecules to increase the adhesion rate of cells, promote gene conduction, inhibit cancer cell development and kill bacteria [33]. In the Tb-loaded MC scaffold, Tb^3+^ can replace Ca^2+^ in the apatite structure and successfully integrate into the collagen, conferring luminescence, photocatalytic and bactericidal activity with good biocompatibility. Castillo *et al.* [83] prepared type I collagen-apatite fibers scaffolds doped with Tb, resulting in composites with long luminescence lifetimes and high relative luminescence intensities. The luminescent properties facilitate the tracking of changes in bone tissue when the scaffold is implanted into the bone defect. Tb^3+^-doped nanocomposites showed good biocompatibility with cell viability higher than 95%. In addition, its effect of promoting osteogenic differentiation is very obvious. Therefore, MC combined with Tb elements provides the possibility to monitor changes in implanted scaffolds and new bone formation *in vivo* in real-time.

## Current status of MC and its application of related absorbable materials

In the process of bone defect repair and bone regeneration, MC can provide good biocompatibility, osteoinductivity and biodegradability, MC also has high porosity, has good hand feeling, is easy to trim and can closely contact the surrounding bone through expansion to increase stability [84, 85]. Therefore, they do not need to perform additional surgical procedures on the surrounding soft tissues when applied, making it widely used in the maxillofacial region, vertebrae, as well as segmental bone defects. The application of MC and its related products in the repair of clinical bone defects is shown in Table 1.

**Table 1.** Clinical applications of MC in bone repair

Implant site	Implant	Characterization	Objective	Results	Conclusions	Ref.
Maxillofacial	ReFit^®^(HOYA Technosurgical, Tokyo, Japan)	A volume ratio of HA to Col 80:20; Porosity 95%; Spongy when wet	Inhibiting the resorption of alveolar bone after tooth extraction	At 3 months after surgery, the alveolar bone height was decreased by 0.00 ± 2.44 mm on the buccal side and 0.35 ± 1.73 mm on the lingual side, while the width was decreased by 1.02 ± 1.64 mm; bone biopsy specimens revealed no remaining implanted material	HA/Col is a reliable material for the restoration of post-extraction alveolar bone	[85]
	The HA/β-TCP + blended purified porcine type I collagen	The weight ratio of purified porcine type I collagen to HA/β-TCP 30:70; the ratio of HA to β-TCP 60:40	Preventing bone resorption when applied to dental sockets immediately after tooth extraction	At 3 months after surgery, a minimal alveolar bone width reduction of 1.03 ± 2.43 mm (*P* < 0.05) was observed, and the height reduction showed a slight decrease to 0.62 ± 1.46 mm (*P* < 0.05); radiographically, the bone height was maintained after 3 months	HA/β-TCP + collagen graft demonstrated adequate safety and efficacy in dental socket preservation following tooth extraction	[86]
	HA/Col (RIFIT; HOYA Co. Ltd., Tokyo, Japan)	Porosity 35%; 100–500 μm pore diameter.; elasticity similar to a water-bearing sponge	To evaluate the utility and efficacy of HA/Col for secondary alveolar bone grafting (ABG)	Compared to cancellous iliac bone graft, the 12-month bone volumes were 0.567 ± 0.066 and 0.596 ± 0.073 ml, without significant difference	The use for ABG of HA/Col produced the same result as an autologous bone	[84]
	MC (Allgens^®^, Beijing Allgens Medical Science and Technology Co., Ltd., China)	Approximately 45% mineral by weight	To evaluate the clinical efficacy of MC for immediate implant placement (IIP) in the esthetic area. The reconstructive treatment protocol was performed 6 months after implantation	At 12 months after surgery, the survival rate of implants was 100%. None of the five parameters of the pink esthetic score (PES), or the total PES (6.07 ± 1.62 versus 6.13 ± 1.41) values were significantly different when comparing the MC and the Bio-Oss group. The percentage of clinical acceptance was 60% in both groups	Application of MC in IIP could achieve a similar effect as Bio-Oss	[87]
	HA/Col (ReFit, HOYA Technosurgical, Tokyo, Japan)	80% HA and 20%Col; 95% porosity; sponge-like characteristics under wet conditions	To clarify whether HA/Col could be useful as a graft material for maxillary sinus floor augmentation (MSFA)	The implant survival rate in this study was 82.4%, and in these cases, the alveolar bone heights, cortical bone thicknesses and values of the implant stability quotient were smaller	If alveolar bone height, cortical bone thickness and healing period are optimized, HA/Col can be a useful graft material for MSFA	[88]
	Coll/Pro Osteon 200	Composed by Avitene Microfibrillar Collagen Hemostat and Granular Pro Osteon 200 coralline HA	To enhance the malar area of patients with inadequate cheekbone projection or facial asymmetry	At 24 months after surgery, the prosthesis seemed to adhere staunchly to the underlying zygomatic bone in all patients, and mature bone was found in 70% of some specimens. At 36 months after surgery, the interface between the prosthesis and bone appeared indistinguishable	The hybrid scaffold seems to be an excellent biomaterial able to drive bone regrowth and remodeling	[89]
Spine	MC-PMMA	The optimal mix ratio of MC particles was 15% by weight	To apply the MC-PMMA bone cement for the treatment of Kümmell disease (KD)	With the MC-PMMA bone cement, the incidence of adjacent vertebral re-fracture was reduced to 10.00%, which was significantly better than that in the traditional bone cement group (38.46%). At 6 months and 1 year of follow-up, the bone mineral density (BMD) of the injured vertebra was significantly higher in the MC-PMMA group than in the traditional group	MC-PMMA bone cement could achieve the same effect as that of PMMA bone cement and was associated with better vertebral height restoration in the long term	[2]
	MC-PMMA	The weight ratio of the powder of bone cement to MC 6:1	To compare the clinical effects and imaging features of PMMA bone cement with and without MC in percutaneous kyphoplasty (PKP) for osteoporotic vertebral compression fractures (OVCF）	The rate of cement leakage and distribution of each vertebra in the MC-PMMA group (6.5%) was less than that of the PMMA group (28.3%). At 12 months after surgery, significant differences were found between the PMMA group (92.2 ± 7.6) and the MC-PMMA group (98.7 ± 7.3). The vertebral height of the MC-PMMA group also decreased. The degree of decline was, however, significantly smaller than that in the PMMA group. And CT findings showed fuzzy boundaries and regenerated bone tissues in the MC-PMMA group	MC-PMMA can form a stable structure in the vertebral body, which can improve the prognosis of patients 1 year after the surgery	[90]
	MC-PMMA	Consisted of 22.6 g of traditional bone cement, 3.4 g of MC and 10 ml of MMA monomer liquid	To analyze the safety and efficacy of PVP with MC-PMMA bone cement for patients aged 80	The MC-PMMA group (3.2%) had a significantly lower incidence of adjacent vertebral re-fracture than the traditional PMMA group (27.9%) (*P* < 0.05). At 12 months, the anterior vertebral height (AVH) of the MC-PMMA group was significantly higher than that of the PMMA group (*P* < 0.01); the IVH of the MC-PMMA group was significantly higher than that of the PMMA group (*P* < 0.05)	MC-PMMA can be used as the first choice for filling material for OVCFs procedures in patients aged 80 and over	[91]
	HA/Col+ bone marrow aspirate (BMA)	The pore size and porosity of the porous HA/Col composite were 100–500 μm, and 95%, 3 ml of BMA was integrated into the HA/Col.	To investigate the effectiveness of HA/Col composite with BMA as a graft substitute in PLIF for the treatment of lumbar spinal diseases	At 12 months after surgery, in the HA/Col, a complete fusion was observed in 38 patients (82.6%), whereas in the local bone graft (LBG), it was observed in 35 patients (76.1%). A complete fusion in the HA/Col increased to 44 patients (95.7%) and 41 patients (89.1%) in the LBG 2 years after the surgery	The HA/Col composite with BMA can be effectively used as an alternative to conventional autologous LBG for intervertebral spinal fusion	[92]
Appendicular skeleton	Collagen/nano-hydroxyapatite composite scaffold (Collapat; Symatese, Chaponost, France)	Composed of a collagen structure in which ceramized nano-hydroxyapatite granules are dispersed	To evaluate the use of collagen/nano-hydroxyapatite composite scaffold on the clinical and functional results of the patient in atrophic non-unions of the femur shaft	The collagen/nano-HA composite scaffold group had a higher healing rate (95.8%) than the control group (93.3%), a significantly faster union time (6.42 ± 1.84 months) than the control group (8.11 ± 2.13), and patients’ psychological well-being and overall health perceptions were at a better level	In the treatment of atrophic non-unions of the femoral shaft isthmic region, the use of collagen/nano-hydroxyapatite composite scaffolds together with intramedullary exchange nailing affects the union positively	[93]
	MC	MC was ground into powders and screened by a 200-mesh sieve	To evaluate clinical outcomes of patients treated with curettage of benign bone tumors and bone grafting with MC	At 12 months after surgery, compared to autologous bone grafts, patients had Lane-Sandhu X-ray scores (11.62 ± 0.506) close to those of controls (11.78 ± 0.428) and Musculoskeletal Tumor Society (MSTS) scores (28.02 ± 1.044) slightly higher than those of controls (26.78 ± 2.184), none of which was statistically different	The artificial biomimetic MC can be used as a good autogenous bone substitute material for the treatment of benign bone tumors	[94]

**Figure 10.**
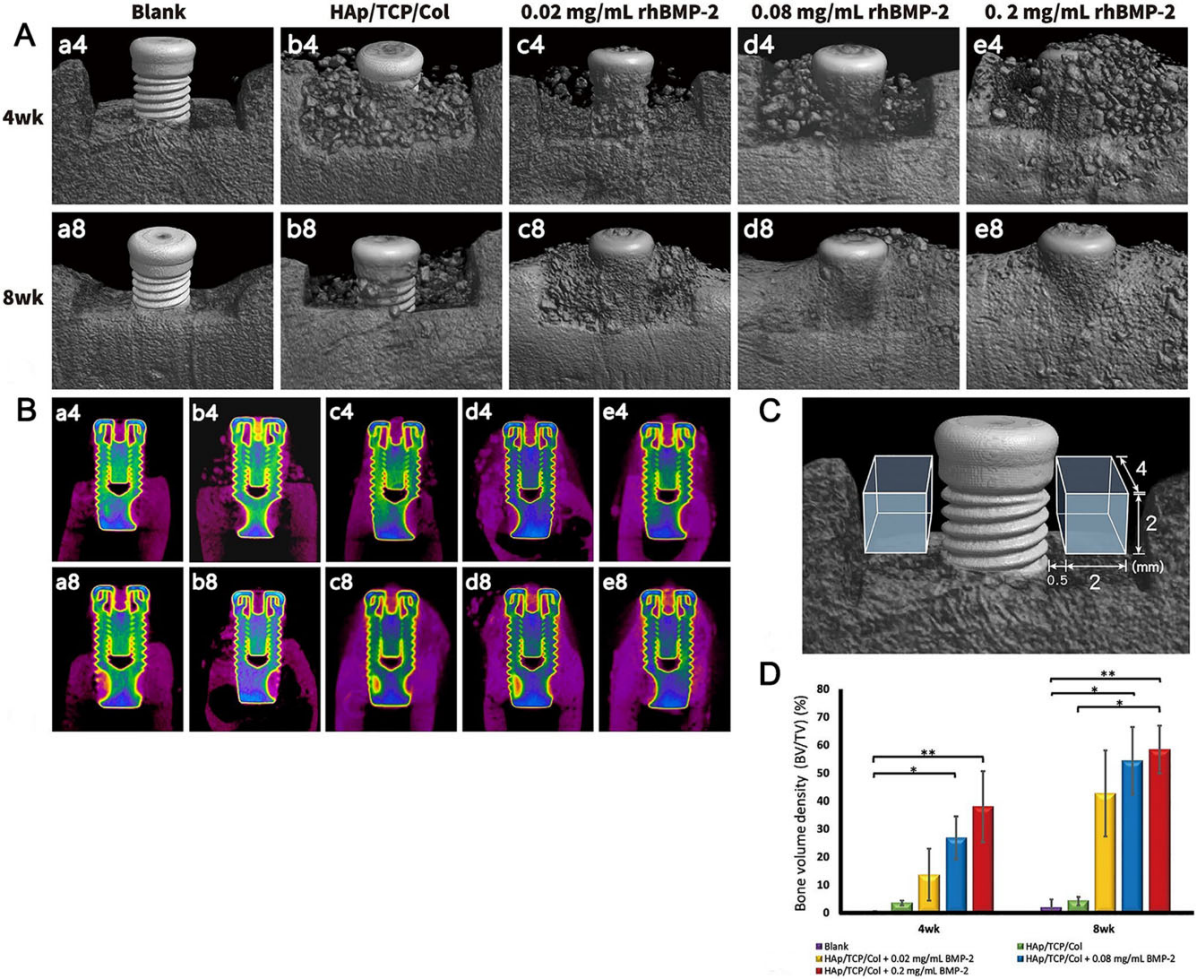
Micro-computed tomography imaging. (**A**) Three-dimensional reconstruction image. 0.08 and 0.2 mg/ml rhBMP-2 restored alveolar bone morphology and dense bone surface at 8 weeks. (**B**) Buccolingual section images. The rhBMP-2 group restored adequate bone height and width for dental implants at Week 8. (**C**) Schematic showing volumes of interest (VOIs) for quantification. (**D**) Morphometric results of new bone within VOIs. At Week 8, bone mineral density (BV/TV) was significantly better in the 0.2 mg/ml rhBMP-2 group than in the control group. Values are presented as mean ± SD. Adjusted *P* < 0.05. Adjusted *P* < 0.01. BV, bone volume; TV, total volume. Copyright 2021, John Wiley.

MC-based implants help to preserve the alveolar bone to prevent and reduce resorption of the alveolar ridge and recession of the interdental papillae, providing the ideal bone height, width and density for subsequent implant placement. Many studies have shown that MC-based composites are biocompatible and safe to use after tooth extraction and can effectively reduce the rate of alveolar bone resorption. Wang *et al.* [95] used self-assembled MC to repair peri-implant bone defects in the mandible of minipigs and found that the scaffold stimulated new bone formation in alveolar ridge reconstruction around dental implants. Chao *et al.* [96] used recombinant bone morphogenetic protein (rhBMP-2) combined with HA/β-tricalcium phosphate (TCP)/collagen (Col) composite to treat alveolar bone defects in beagle dogs. As shown in Fig. 10, rhBMP-2 combined with HA/TCP/Col at a concentration of 0.2 mg/ml was found to complete bone repair of large defects around implants within 8 weeks. Therefore, self-assembled MC and HA/TCP/Col composites combined with 0.2 mg/ml rhBMP-2 have the prospect of application in clinical practice.

**Figure 11.**
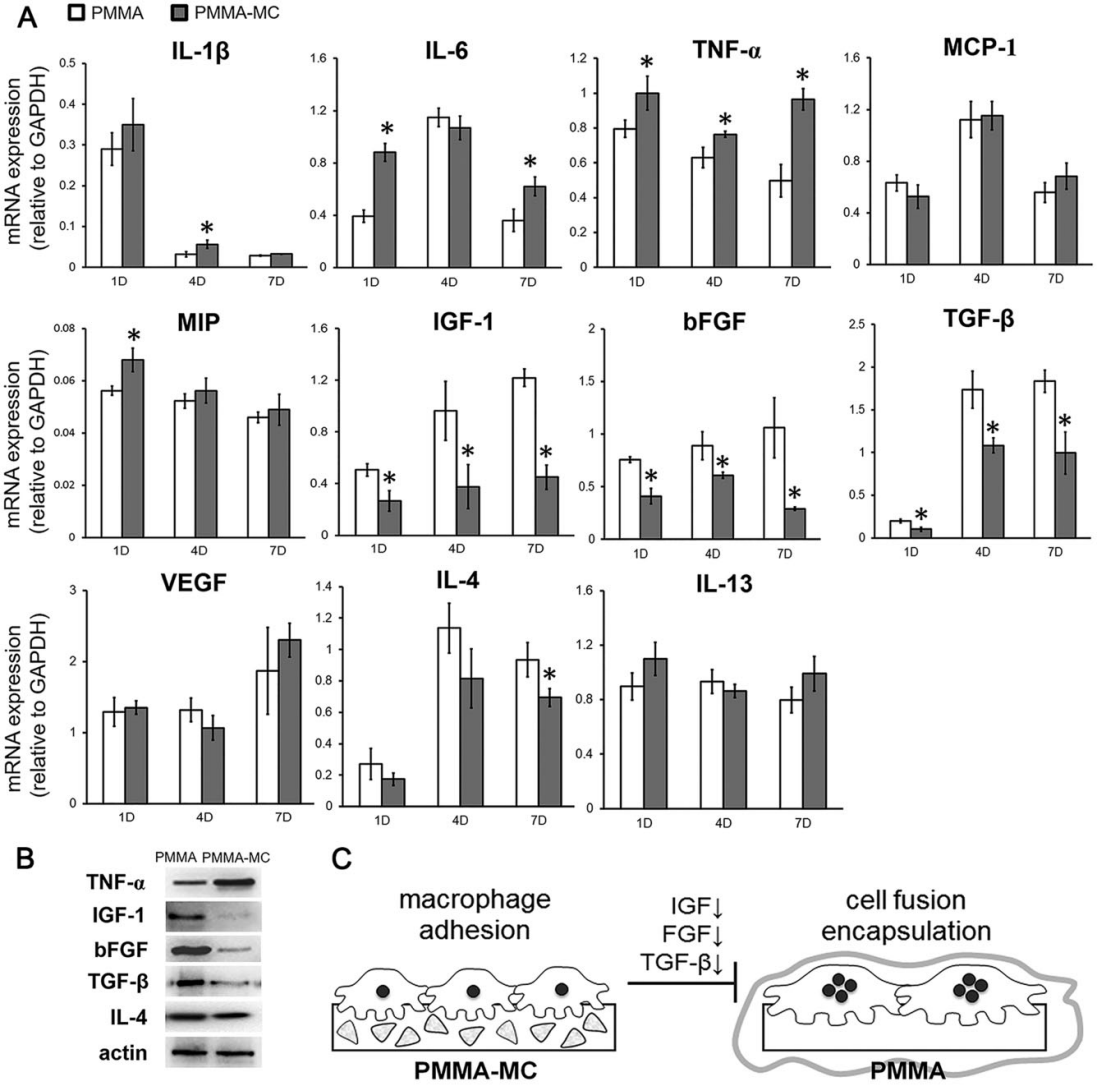
MC-PMMA regulates the expression of related growth factors. (**A**) Gene expression levels of proinflammatory cytokines, chemokines, fibroblast stimulating growth factor and macrophage fusion-related genes at 1, 4 and 7 days of culture between PMMA or PMMA-MC materials and co-macrophages by the qRT–PCR analysis were significantly different compared with the PMMA group (*P* < 0.05). (**B**) Western blotting confirmation of the differentially expressed genes. (**C**) The schematic illustration shows the influence of MC particles on the fibrous encapsulation of PMMA. Copyright 2019, Oxford University Press.

Polymethyl methacrylate (PMMA) bone cement is widely used in the treatment of spine-related diseases. However, PMMA still has many shortcomings, such as a lack of osseointegration ability, increased risk of adjacent vertebral fractures and recompression fractures after surgery, and even cardiopulmonary embolism [97, 98]. Li *et al.* [99] found that the addition of MC decreased the elastic modulus of PMMA from 1.91 ± 0.08 GPa to 1.20 ± 0.12 GPa. The osteogenic activity of MC-PMMA promotes the integration of the cement-bone interface. Yang *et al.* [3] found that MC-PMMA could hinder the proliferation and fusion of macrophages, and significantly down-regulate the expression of fibroblast growth factors, insulin-like growth factor and basic fibroblast growth factor, as shown in Fig. 11, which contributed to the direct contact between the scaffold and new bone. MC-PMMA and PMMA cement were injected into a goat model of disk degeneration. The results showed that the circumferential contact index between MC-PMMA and bone was 36.4% higher, and histological staining revealed that the surface of MC-PMMA was in direct contact with new bone, while PMMA was encapsulated by fibrous tissue. Kong *et al.* [100] developed a minimally invasive injectable lumbar interbody fusion goat model using MC-PMMA. The osteogenic potential of MC-PMMA, PMMA and titanium cage filled with autogenous bone (TC-AB) was compared at 3 and 6 months after surgery. The results showed that MC-PMMA had good osteogenic potential, osteogenic induction and osteointegration. Therefore, MC-PMMA bone cement has profound clinical application value in the treatment of spine-related diseases. In addition, Tamaddon *et al.* [101] demonstrated that HA/Col combined with autologous bone marrow concentrates (autologous bone marrow concentrates) (BMC) scaffolds have the potential to be applied in the repair of clinical appendicular bone (skeleton) defects.

Research into the use of MC for the repair of bone defects is currently in full swing; however, there are few commercially available MC products. Table 2 shows some MC-based bone replacement products in recent years. MC-based products are commonly used in non-load-bearing areas, such as the maxillofacial skeleton, e.g. ReFit^®^ (HOYATechnosurgical, Tokyo, Japan) with its sponge-like properties allows perfect contact with surrounding bone of the extraction socket, it is a reliable material for preserving alveolar bone after tooth extraction [85]. However, this effect only provides reliable results at the upper anterior, lower premolar and lower molar, but not at the upper premolar and molar, and the reason may be its wide socket size at the posterior region. MC is also used in the repair of appendicular skeleton defects. Nevzat *et al.* [93] evaluated the clinical and functional outcomes of using a collagen/n-HA composite scaffold for atrophic bone non-union, and showed that the composite scaffold improved the healing rate of atrophic bone non-union of the femoral stem. Therefore, MC can be widely used in the repair of bone defects.

**Table 2.** MC-based bone substitute products

Trade/device name	Owner	Composition	Physical form	Intended use	Approval time
Mineral collagen composite bioactive moldable	Collagen Matrix, Inc., USA	Calcium phosphate Bioactive glass Type I bovine collagen	Strip or cylindrical matrix	Bone void filler for voids or gaps	11/2022
Porcine mineral collagen composite	Collagen Matrix, Inc., USA	Anorganic bone mineral Type I collagen	Preformed sponge matrix	Bone grafting in periodontal, oral and maxillofacial surgeries	03/2021
Porcine mineral collagen composite moldable	Collagen Matrix, Inc., USA	Anorganic (porcine) bone mineral Purified (porcine) collagen	Block shaped	Bone grafting in periodontal, oral and maxillofacial surgeries	07/2020
Bioactive bone graft putty	BioStructures, LLC., USA	Calcium phosphate (60%HA: 40%TCP) Bioactive glass Alkylene oxide polymer	Putty	Bone void filler for voids or gaps	01/2014
Vitoss foam bone graft substitute	Orthovita, Inc., USA	Calcium phosphate Bioactive glass Type I bovine collagen	Strip or cylindrical matrix	Bone void filler for voids or gaps	11/2008

Despite the many applications of MC in bone defect repair, there are still many problems. For example, MC scaffold implantation at the site of a bone defect does not have a significant early bone repair effect, Gao *et al.* [94] used MC for bone repair after scraping of benign bone tumors, and at 1 month postoperatively, the osteogenic outcome of the MC group was lower than that of autologous bone grafts. The reason may be that autologous bone contains osteoblasts and growth factors, while MC requires the growth of osteoblasts and the delivery of growth factors. Therefore, osseointegration of BMC and growth factors and mesenchymal stem cells may be a promising approach to enhance the early osteogenic effects of scaffolds.

In addition, Jones *et al.* [102] compared the physicochemical properties and biocompatibility of OsteoGen^®^ Bioactive Resorbable Calcium Apatite, 90% Geistlich Bio-Oss^®^ granules with the addition of 10% porcine collagen and natural bone. The cells were found to prefer the larger pore size, superiority and higher interconnectivity of natural bone compared to both scaffolds. Besides, highly mineralized scaffolds slow down cell proliferation and osteogenic function in promoting bone growth, possibly due to the fact that cells do not attach easily to mineral components. Therefore, the MC scaffold with the appropriate pore size, porosity and degree of mineralization still requires continuous research.

The ideal load-bearing bone repair material should be strong enough to function on its own. However, highly porous MC scaffolds have sponge-like properties in wet conditions [85], so they are rarely used on their own and often require fixation aids such as metal plates.

Besides, MC poses a risk of infection in bone defect repair. Therefore, patients with diseases, such as osteomyelitis, bone tuberculosis, maxillary sinusitis, runny nose and periodontal disease, should be avoided for the application of MC in bone defects [84]. Stent preparation, transport procedures and surgical procedures should be strictly aseptic. In addition, the combination of MC and antibiotics may also reduce the risk of infection during bone repair.

## Conclusion and perspective

MC scaffold of bone tissue engineering is a new and safe bone repair material composed of collagen and HA, which can simulate the chemical and physical complexity of natural bone, and its micro/nanostructure is very close to the extracellular matrix of natural bone tissue [103], and it can also provide biological activity guidance signals. MC and other related absorbable materials gradually degrade *in vivo*, while bone tissue grows into the scaffold, the mechanical properties of the scaffold gradually decrease, and the biological stress is transferred from the graft material to the new bone tissue, promoting bone regeneration while avoiding stress shielding [104]. MC not only has its own good bone repair ability, but also can be combined with polymer materials, metal elements, inorganic materials and so on [45, 105, 106], and can also be equipped with drugs that promote vascular growth and bone regeneration and jointly play an osteogenic role.

Metal elements are important components in the fields of electrochemistry, biotechnology and medicine and play an important role in the field of bone regeneration. Local delivery of therapeutic ions to the lesion is an ideal treatment modality. MC scaffolds compounded with inorganic metal elements are dissolved and released at the implantation site and stimulate osteogenesis and angiogenesis of the associated cells. Osseointegration of elements, such as Mg, Zn and Sr, with MC has performed well *in vitro*, *in vivo* animal experiments, and in clinical studies. It not only optimizes the physical and chemical structure of composite scaffolds, but also promotes osteogenic chemical signal transduction and related gene up-regulation, promotes osteogenesis and angiogenesis and has the potential to be an ideal bone defect repair material.

Currently, the experimental research and clinical application of MC and inorganic metal elements have been very extensive, and great progress has been made. However, the application of osseointegration of MC with a wider range of metal elements in bone repair is more expected, such as copper [107], which can promote the angiogenesis of bone tissue, cobalt [108], yttrium [109], which can improve the mechanical properties of the complex, and niobium [110], which can enhance osteoblast activity and mineralization. In addition to its application in bone, the application of MC composite metal element materials in soft tissue and organ regeneration is also worth exploring. Artificial bone was constructed by metal, natural/synthetic polymer and bioceramics, combined with growth factors and other composite scaffolds to meet the therapeutic goals of angiogenesis, osteoinduction, bone conduction and appropriate mechanical environment, which is worthy of more and further study.
